# *eNOS* rs2070744 polymorphism might influence predisposition to hemorrhagic cerebral vascular diseases in East Asians: A meta‐analysis

**DOI:** 10.1002/brb3.1538

**Published:** 2020-03-26

**Authors:** Qiuling Wang, Hongri Sun, Xiaoguang Qi, Minfeng Zhou

**Affiliations:** ^1^ Department of Nursing Changyi Peoples Hospital Changyi China; ^2^ Department of Neurology Changyi Peoples Hospital Changyi China; ^3^ Department of General Surgery Changyi Peoples Hospital Changyi China; ^4^ Department of General practice Zhuji Affiliated Hospital of Shaoxing University Zhuji China

**Keywords:** East Asians, endothelial nitric oxide synthase (*eNOS*), gene polymorphisms, hemorrhagic cerebral vascular diseases, meta‐analysis

## Abstract

**Introduction:**

Endothelial nitric oxide synthase (*eNOS*) polymorphisms might influence predisposition to hemorrhagic cerebral vascular diseases, but the results of already published studies regarding relationship between *eNOS* polymorphisms and hemorrhagic cerebral vascular diseases were still controversial.

**Methods:**

The authors performed this meta‐analysis to estimate relationship between *eNOS* polymorphisms and hemorrhagic cerebral vascular diseases in a larger pooled population by combing the results of already published related studies. The authors searched Pubmed, Embase, Web of Science, and CNKI for already published studies.

**Results:**

Eighteen already published studies were pooled analyzed in this meta‐analysis. The pooled meta‐analyses results showed that *eNOS* rs2070744 polymorphism was significantly associated with predisposition to hemorrhagic cerebral vascular diseases in East Asians (dominant comparison: OR = 0.77, *p* = .01; overdominant comparison: OR = 1.24, *p* = .04; allele comparison: OR = 0.78, *p* = .006) Nevertheless, the pooled meta‐analyses did not reveal any positive results for *eNOS* rs1799983 and rs869109213 polymorphisms.

**Conclusions:**

This meta‐analysis suggested that *eNOS* rs2070744 polymorphism, but not rs1799983 and rs869109213 polymorphisms, might influence predisposition to hemorrhagic cerebral vascular diseases in East Asians.

## Graphical abstract

This is a comprehensive meta‐analysis about *eNOS* polymorphisms and hemorrhagic cerebral vascular diseases. We found that *eNOS* rs2070744 polymorphism might influence predisposition to hemorrhagic cerebral vascular diseases in East Asians. Our findings indicated that *eNOS* may involve in the development of hemorrhagic cerebral vascular diseases.

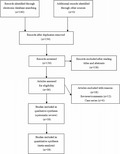

## 1 INTRODUCTION

Hemorrhagic cerebral vascular disease, characterized by structural or functional abnormalities of cerebral vessels, and associated spontaneous bleeding into brain tissue, is one of the leading causes of death and disability all over the world (Global Burden of Disease Study 2013 Collaborators, 2015; Caceres & Goldstein, 2012). Although its definite etiologies and pathogenesis mechanisms are still unclear, accumulating evidence suggested that genetic architecture greatly influence its development. First, the incidences of hemorrhagic cerebral vascular diseases in different populations differed significantly (Backhaus et al., 2015; Krishnan et al., 2018), and genetic background was probably the underlying reason of this phenomenon. Second, previous association studies also detected numerous predisposing gene loci of hemorrhagic cerebral vascular diseases in different populations (Carpenter, Singh, Gandhi, & Prestigiacomo, 2016; Chen, Chang, & Chen, 2018). However, the etiologies and pathogenesis mechanisms of hemorrhagic cerebral vascular diseases are extremely sophisticated, and genetic factors that contribute to the development of hemorrhagic cerebral vascular diseases still need intensively explorations.

Endothelial nitric oxide synthase (eNOS) is essential for maintaining vascular homeostasis and plays vital roles in modulating vascular endothelial function (Besler et al., 2011; Tsutsui, Shimokawa, Morishita, Nakashima, & Yanagihara, 2006). Therefore, if a polymorphism can impact gene expression or protein structure of *eNOS*, it is likely that this polymorphism might lead to severe vascular endothelial dysfunction and influence predisposition to hemorrhagic cerebral vascular diseases.

In the last two decades, investigators across the world extensively explored relationship between *eNOS* polymorphisms and hemorrhagic cerebral vascular diseases, especially for intracranial aneurysm (IA) and its associated aneurysmal subarachnoid hemorrhage (aSAH), yet the relationship between *eNOS* polymorphisms and hemorrhagic cerebral vascular diseases is still controversial. Thus, we performed this meta‐analysis to get a more statistically reliable conclusion regarding relationship between *eNOS* polymorphisms and hemorrhagic cerebral vascular diseases by pooling the results of already published relevant studies.

## 2 MATERIALS AND METHODS

The PRISMA guideline was followed by the authors when conducting this meta‐analysis (Moher, Liberati, Tetzlaff, Altman, & PRISMA group, 2009).

### 2.1 Literature search and inclusion criteria

Literature searching of Pubmed, Web of Science, Embase, and CNKI was performed by the authors using the following terms: (endothelial nitric oxide synthase or nitric oxide synthase type III or eNOS or NOS3) and (polymorphism or variant or variation or mutation or SNP or genome‐wide association study or genetic association study or genotype or allele) and (hemorrhagic cerebral vascular disease or hemorrhagic cerebrovascular disease or cerebral hemorrhage or basal ganglia hemorrhage or putaminal hemorrhage or subarachnoid hemorrhage or cerebrum hemorrhage or brain hemorrhage or intracranial hemorrhage or intracranial aneurysm or cerebral aneurysm). When searching CNKI, the authors translated searching terms into Chinese. The authors also checked the references of obtained articles manually for additional related studies.

Eligible studies must meet all of three inclusion criteria: I. formally published case–control studies evaluating relationship between *eNOS* polymorphisms and hemorrhagic cerebral vascular diseases; II. provide sufficient genotypic data of *eNOS* polymorphisms in patients with hemorrhagic cerebral vascular diseases and controls; III. the whole manuscript is available in English or Chinese. Articles were excluded when at least one of the following three conditions was fulfilled: I. studies not concerning *eNOS* polymorphisms and hemorrhagic cerebral vascular diseases; II. reviews or expert comments; III. case series only involved participants with hemorrhagic cerebral vascular diseases. When duplicate reports were observed during literature searching, only the most recent one was included for pooled meta‐analyses. The literature searching was performed by the first author and the second author, and the latest literature searching update was performed on 10 November 2019.

### 2.2 Data extraction and quality assessment

We extracted following items from included studies: I. surname of the first author; II. year of online publication; III. country and ethnicity of involved participants; IV. number of patients and controls; V. genotypic distributions of *eNOS* polymorphisms in patients and control subjects. We also calculated *p* values of Hardy–Weinberg equilibrium (HWE) based on genotypic distributions of *eNOS* polymorphisms using chi‐square test.

The authors used Newcastle–Ottawa scale (NOS) to assess the quality of included studies (Stang, 2010). The NOS's score range is from zero to nine, and the methodology quality of an article was considered to be good when it got a score of more than seven.

Data extraction and quality assessment of included studies were performed by the first author and the second author separately. The authors wrote to the corresponding authors for additional data when they failed to extract necessary information from included studies. In case of disagreement between the first author and the second author, the two authors would consult the corresponding author for suggestions, and a consensus must be reached among three authors.

### 2.3 Statistical analyses

The authors used Review Manager to pool meta‐analyses results (Version 5.3.3, The Cochrane Collaboration, Software Update). The authors calculated ORs and 95% CIs of *Z* test to evaluate relationship between *eNOS* polymorphisms and predisposition to hemorrhagic cerebral vascular diseases in dominant, recessive, overdominant, and allele models (Keat Wei, Griffiths, Irene, & Kooi, 2019; Wei, Griffiths, Kooi, & Irene, 2019). The authors set the statistical significant threshold at 0.05. The authors used *I* ^2^ statistics to estimate heterogeneity. *I* ^2^ values of 25%, 50%, and 75% represented low, moderate, and high heterogeneities, respectively. The authors used DerSimonian–Laird method (random‐effect model) to pool the results if *I* ^2^ is larger than 50%. Otherwise, the authors used Mantel–Haenszel method (fixed‐effect model) to pool the results. The authors also conducted subgroup analyses by ethnicity. The authors examined stabilities of pooled results through omitting one study each time and pooling the results of the other studies. The authors examined publication biases through funnel plots.

## 3 RESULTS

### 3.1 Characteristics of included studies

**Figure 1:**
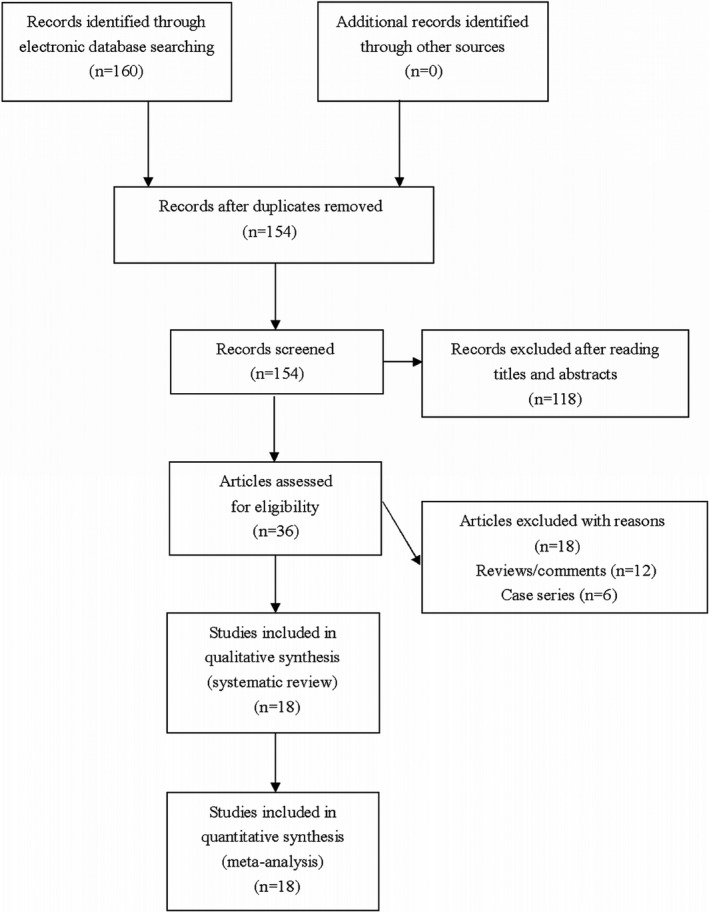
Flowchart of study selection for this meta‐analysis

One hundred and sixty articles were retrieved by the authors through our literature searching strategy. The authors assessed thirty‐six articles for eligibility after omitting unrelated or repeated reports. Twelve reviews and six case series were further excluded by the authors. Totally eighteen studies were finally pooled in our meta‐analyses (Figure 1). Extracted data of eligible studies were summarized in Table 1.

**Table 1:** The characteristics of included studies in this meta‐analysis

First author (year)	Country	Ethnicity	Type of disease	Sample size	Genotype distribution		*p* value for HWE	NOS score
					Cases	Controls		
rs1799983 G/T					GG/GT/TT			
Du (2014)	China	East Asian	Aneurysmal subarachnoid hemorrhage	60/60	27/19/14	39/16/5	.097	7
Khurara (2004)	USA	Caucasian	Aneurysmal subarachnoid hemorrhage	51/90	20/25/6	20/55/15	.032	7
Kim (2011)	Korea	East Asian	Intracranial aneurysms	149/121	125/24/0	98/23/0	.248	8
Konar (2019)	India	Mixed	Aneurysmal subarachnoid hemorrhage	100/100	71/23/6	76/23/1	.607	7
Koshy (2008)	India	Mixed	Aneurysmal subarachnoid hemorrhage	122/224	85/35/2	159/61/4	.501	8
Krex (2006)	Germany	Caucasian	Intracranial aneurysms	142/190	64/67/11	96/76/18	.602	7
Krischek (2006)	Japan	East Asian	Intracranial aneurysms	405/176	349/50/6	145/23/8	<.001	8
Liu (2013)	China	East Asian	Intracranial aneurysms	82/82	49/25/8	36/38/8	.656	8
Ozum (2008)	Turkey	Caucasian	Aneurysmal subarachnoid hemorrhage	53/60	26/23/4	34/22/4	.863	8
Staalsø (2014)	Denmark	Caucasian	Aneurysmal subarachnoid hemorrhage	331/498	151/153/27	233/216/49	.918	8
Xu (2009)	China	East Asian	Aneurysmal subarachnoid hemorrhage	58/67	27/17/14	45/15/7	.005	7
Zhao (2008)	China	East Asian	Hypertensive intracerebral hemorrhage	70/112	53/15/2	105/7/0	.732	7
Zhe (2019)	China	East Asian	Aneurysmal subarachnoid hemorrhage	169/156	90/50/29	106/38/12	.003	8
rs2070744 T/C					TT/TC/CC			
Akagawa (2005 a)	Japan	East Asian	Intracranial aneurysms	220/214	176/41/3	179/34/1	.648	8
Akagawa (2005 b)	Korea	East Asian	Intracranial aneurysms	191/191	144/46/1	149/41/1	.304	8
Deng (2018)	China	East Asian	Aneurysmal subarachnoid hemorrhage	71/142	70/1/0	130/11/1	.177	7
Khurara (2003)	USA	Caucasian	Aneurysmal subarachnoid hemorrhage	52/90	12/35/5	28/46/16	.699	7
Kim (2011)	Korea	East Asian	Intracranial aneurysms	149/121	122/24/3	99/22/0	.271	8
Konar (2019)	India	Mixed	Aneurysmal subarachnoid hemorrhage	100/100	71/23/6	76/23/1	.607	7
Koshy (2008)	India	Mixed	Aneurysmal subarachnoid hemorrhage	122/224	68/51/3	136/81/7	.219	8
Krex (2006)	USA	Caucasian	Intracranial aneurysms	135/184	48/60/27	71/86/27	.908	7
Krischek (2006)	Japan	East Asian	Intracranial aneurysms	405/176	326/72/7	145/23/8	<.001	8
Liu (2013)	China	East Asian	Intracranial aneurysms	82/82	59/19/4	69/12/1	.569	8
Song (2006)	Korea	East Asian	Aneurysmal subarachnoid hemorrhage	132/113	106/26/0	100/13/0	.516	7
Staalsø (2014)	Denmark	Caucasian	Aneurysmal subarachnoid hemorrhage	331/498	145/147/39	197/233/68	.946	8
Zhe (2019)	China	East Asian	Aneurysmal subarachnoid hemorrhage	169/156	82/60/27	95/47/14	.029	8
rs869109213 VNTR					4b4b/4b4a/4a4a			
Bi (2010)	China	East Asian	Aneurysmal subarachnoid hemorrhage	80/107	59/18/3	91/16/1	.753	8
Fang (2011)	China	East Asian	Hypertensive intracerebral hemorrhage	62/236	52/10/0	187/48/1	.257	7
Khurara (2004)	USA	Caucasian	Aneurysmal subarachnoid hemorrhage	51/90	25/25/1	70/16/4	.029	7
Kim (2011)	Korea	East Asian	Intracranial aneurysms	149/121	122/24/3	96/25/0	.205	8
Konar (2019)	India	Mixed	Aneurysmal subarachnoid hemorrhage	100/100	65/30/5	72/25/3	.648	7
Koshy (2008)	India	Mixed	Aneurysmal subarachnoid hemorrhage	122/224	77/40/5	143/77/4	.077	8
Krex (2006)	Germany	Caucasian	Intracranial aneurysms	142/189	98/41/3	126/55/8	.525	7
Krischek (2006)	Japan	East Asian	Intracranial aneurysms	405/176	325/70/10	143/30/3	.341	8
Staalsø (2014)	Denmark	Caucasian	Aneurysmal subarachnoid hemorrhage	332/498	254/71/7	344/145/9	.155	8

Abbreviations: HWE, Hardy–Weinberg equilibrium; NA, not available; NOS, Newcastle–ottawa scale.

### 3.2 Meta‐analyses of *eNOS* rs1799983 polymorphism and hemorrhagic cerebral vascular diseases

Thirteen studies involving 1792 cases and 1936 control subjects were eligible for estimation of relationship between *eNOS* rs1799983 polymorphism and hemorrhagic cerebral vascular diseases. The pooled meta‐analyses did not reveal any significant associations for *eNOS* rs1799983 polymorphism and hemorrhagic cerebral vascular diseases (see Table 2).

**Table 2:** Pooled meta‐analyses results of the current study

Polymorphisms	Population	Sample size	Dominant comparison		Recessive comparison		Overdominant comparison		Allele comparison	
			*p* value	OR (95%CI)	*p* value	OR (95%CI)	*p* value	OR (95%CI)	*p* value	OR (95%CI)
rs1799983 G/T	Overall	1792/1936	.21	0.84 (0.64–1.10)	.33	1.26 (0.80–1.98)	.24	1.09 (0.94–1.27)	.14	0.83 (0.64–1.06)
	Caucasian	577/838	.71	0.96 (0.78–1.19)	.26	0.81 (0.56–1.17)	.30	1.12 (0.90–1.39)	.82	1.02 (0.87–1.20)
	East Asian	993/774	.20	0.71 (0.42–1.20)	.20	1.66 (0.76–3.61)	.55	1.13 (0.76–1.68)	.15	0.69 (0.42–1.14)
	aSAH	944/1255	.10	0.77 (0.56–1.05)	.10	1.57 (0.92–2.70)	.24	1.11 (0.93–1.34)	.06	0.75 (0.55–1.01)
	IA	848/681	.85	0.95 (0.55–1.64)	.32	0.77 (0.46–1.29)	.75	1.09 (0.65–1.83)	.89	0.97 (0.60–1.56)
rs2070744 T/C	Overall	2159/2291	.05	0.87 (0.76–1.00)	.47	1.10 (0.85–1.42)	.10	1.12 (0.98–1.29)	.06	0.90 (0.80–1.00)
	Caucasian	518/772	.69	1.05 (0.83–1.32)	.71	0.94 (0.68–1.30)	.89	0.98 (0.79–1.23)	.64	1.04 (0.88–1.22)
	East Asian	1419/1195	**.01**	**0.77 (0.63–0.94)**	.16	1.42 (0.87–2.32)	**.04**	**1.24 (1.01–1.52)**	**.006**	**0.78 (0.66–0.93)**
	aSAH	977/132	.21	0.82 (0.59–1.12)	.86	1.03 (0.75–1.40)	.28	1.11 (0.92–1.33)	.27	0.85 (0.64–1.31)
	IA	1182/968	.09	0.83 (0.67–1.03)	.30	1.27 (0.81–2.01)	.21	1.15 (0.93–1.42)	.06	0.84 (0.70–1.01)
rs869109213 VNTR	Overall	1443/1741	.49	0.90 (0.66–1.22)	.26	1.32 (0.81–2.14)	.65	1.08 (0.78–1.48)	.40	0.90 (0.70–1.15)
	Caucasian	525/777	.62	0.82 (0.37–1.82)	.48	0.77 (0.37–1.61)	.53	1.34 (0.54–3.32)	.79	0.93 (0.53–1.63)
	East Asian	696/640	.72	0.95 (0.71–1.27)	.12	2.19 (0.82–5.86)	.83	0.97 (0.72–1.31)	.39	0.89 (0.68–1.16)
	aSAH	685/1019	.24	0.72 (0.41–1.25)	.21	1.48 (0.80–2.75)	.33	1.33 (0.74–2.40)	.19	0.75 (0.49–1.15)
	IA	758/722	.53	1.09 (0.84–1.42)	.81	1.10 (0.50–2.41)	.49	0.91 (0.69–1.19)	.60	1.07 (0.84–1.35)

Abbreviations: aSAH, aneurysmal subarachnoid hemorrhage; CI, confidence interval; IA, intracranial aneurysm; OR, odds ratio.

The values in bold represent there is statistically significant differences between cases and controls.

### 3.3 Meta‐analyses of *eNOS* rs2070744 polymorphism and hemorrhagic cerebral vascular diseases

Twelve studies involving 2,159 cases and 2,291 control subjects were eligible for estimation of relationship between *eNOS* rs2070744 polymorphism and hemorrhagic cerebral vascular diseases. *eNOS* rs2070744 polymorphism was found to be significantly associated with hemorrhagic cerebral vascular diseases in East Asians (dominant comparison: OR = 0.77, 95% CI 0.63–0.94; overdominant comparison: OR = 1.24, 95% CI 1.01–1.52; allele comparison: OR = 0.78, 95% CI 0.66–0.93) (see Table 2).

### 3.4 Meta‐analyses of *eNOS* rs869109213 polymorphism and hemorrhagic cerebral vascular diseases

Nine studies involving 1,443 cases and 1741 control subjects were eligible for estimation of relationship between *eNOS* rs869109213 polymorphism and hemorrhagic cerebral vascular diseases. The pooled meta‐analyses did not reveal any significant associations for *eNOS* rs869109213 polymorphism and hemorrhagic cerebral vascular diseases (see Table 2).

### 3.5 Sensitivity analyses

Stabilities of pooled meta‐analyses results were examined by omitting one study each time and pooling the results of the other studies. The trends of associations remained unchanged in sensitivity analyses, indicating that our pooled meta‐analyses results were statistically stable.

### 3.6 Publication biases

Publication biases were examined by funnel plots. Funnel plots were overall symmetrical, suggesting that our pooled meta‐analyses results were not likely to be severely influenced by publication biases (Figure S1).

## 4 DISCUSSION

Endothelial nitric oxide synthase, which is primarily found in the vascular endothelium, can continuously generate NO, and thus, it is essential for maintaining basal vascular tone and normal cerebral blood flow, and its dysregulation has also been found to be closely related to vascular endothelial dysfunction (Rudic et al., 1998; Faraci & Brian 1994). Since vascular endothelial dysfunction is clearly implicated in vascular diseases, *eNOS* gene polymorphisms were also intensively studied regarding their relationship with predisposition to various types of cerebral vascular diseases. There were already abundant meta‐analyses regarding *eNOS* gene polymorphisms and ischemic cerebral vascular diseases (Shyu et al., 2017; Wei et al., 2017). So in this meta‐analysis, we focused on hemorrhagic cerebral vascular diseases. The pooled meta‐analyses results demonstrated that *eNOS* rs2070744 polymorphism might influence susceptibility to hemorrhagic cerebral vascular diseases in East Asians, but we failed to find any significant associations for *eNOS* rs1799983 and rs869109213 polymorphisms. The trends of associations remained unchanged in sensitivity analyses, suggesting that our pooled meta‐analyses results were quite statistically stable.

Few points need to be considered when interpreting our findings. First, previous experimental studies demonstrated that all investigated polymorphisms were correlated with altered gene expression or protein structure of eNOS (AlFadhli, 2013; Dosenko, Zagoriy, Haytovich, Gordok, & Moibenko, 2006; Elakkad, Abou‐Aisha, Hassanein, & Gad, 2017). To be brief, the rs2070744 polymorphism is associated with a thymine to cytosine mutation at coden‐786 in the 5′‐flanking region of the *eNOS* gene, which can significantly reduce *eNOS* gene expression. The rs1799983 polymorphism is associated with a Glu‐to‐Asp change at nucleotide 298 in exon 7 that is demonstrated to be associated with a trend of reduction of eNOS enzyme activity, and rs869109213 polymorphism, which can result in a variable number of tandem repeats (27 bp VNTRs) in intron 4, is also associated with altered eNOS enzyme activity. Thus, it is likely that these *eNOS* variations might influence normal functioning of eNOS, lead to vascular endothelial dysfunction, and influence predisposition to hemorrhagic cerebral vascular diseases. In this meta‐analysis, only rs2070744 polymorphism was found to be significantly associated with predisposition to hemorrhagic cerebral vascular diseases. Nevertheless, we noticed that the trends of associations for rs1799983 and rs869109213 polymorphisms were actually similar to rs2070744 polymorphism. Therefore, maybe our pooled meta‐analyses were still not statistically sufficient to detect the real associations between *eNOS* polymorphisms and hemorrhagic cerebral vascular diseases, and future studies in larger populations are still needed so as to get a more statistically robust conclusion. Second, the etiologies and pathogenesis mechanisms of hemorrhagic cerebral vascular diseases are extremely sophisticated. In this meta‐analysis, we noticed that almost all eligible studies were about IA and its associated aSAH, whereas only a few studies were about hypertensive cerebral hemorrhage. Nevertheless, considering that the majority of hemorrhagic cerebral vascular diseases should be related to hypertensive disorders. Future studies should concentrate more on elucidating the relationship between *eNOS* polymorphisms and hemorrhagic cerebral vascular diseases of other etiologies, particularly hypertension. Third, we aimed to analyze all *eNOS* polymorphisms at the beginning. However, we did not find sufficient eligible studies to support meta‐analyses of other *eNOS* polymorphisms, so we have to focus on only three polymorphisms in this meta‐analysis.

Like all meta‐analyses, a few limitations of our pooled meta‐analyses have to be acknowledged. First, our pooled meta‐analyses results were derived from pooling unadjusted findings of eligible studies since the authors did not have raw data (Xie, Shi, & Liu, 2017). Second, environmental factors might also influence relationship between *eNOS* polymorphisms and hemorrhagic cerebral vascular diseases. However, most investigators only focused on genetic associations in their works, so genetic–environmental interactions could not be explored in this meta‐analysis (Wang et al., 2018). Third, we did not consider gray literatures (data that are not formally published in scientific books or journals, which include working papers, theses and dissertations, market reports, government documents, and conference posters). Therefore, despite that funnel plots were overall symmetrical, we still could not rule out the possibility that our pooled results might be affected by potential publication biases (Suvatha et al., 2018).

So to conclude, this meta‐analysis demonstrated that *eNOS* rs2070744 polymorphism might influence predisposition to hemorrhagic cerebral vascular diseases in East Asians. These results also suggested that *eNOS* might involve in the development of hemorrhagic cerebral vascular diseases, and it may serve as a potential therapeutic target for hemorrhagic cerebral vascular diseases.

## CONFLICT OF INTEREST

The authors declare that they have no conflict of interest.

## AUTHORS' CONTRIBUTIONS

Qiuling Wang and Minfeng Zhou designed this meta‐analysis. Qiuling Wang and Hongri Sun searched literatures. Xiaoguang Qi analyzed the data. Qiuling Wang and Minfeng Zhou wrote the manuscript. All authors approved the final manuscript as submitted.

## ETHICAL APPROVAL

This article does not contain any studies with human participants or animals performed by any of the authors; thus, ethical approval is not required.
