# Supplementary material for: eNOS rs2070744 polymorphism might influence predisposition to hemorrhagic cerebral vascular diseases in East Asians: A meta‐analysis
Source: Brain Behav. 2020 Mar 26;10(5):e01538. doi: 10.1002/brb3.1538 (PMC7218252; doi:10.1002/brb3.1538)
Supplement: Supplementary file 1 [file BRB3-10-e01538-s001.docx]

Funnel plots of investigated polymorphisms


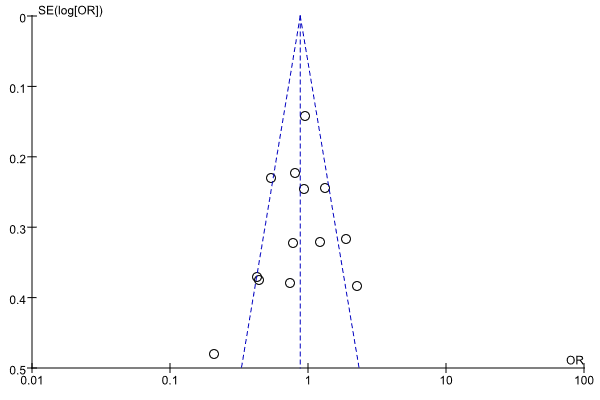


Funnel plot of rs1799983 polymorphism and hemorrhagic cerebral vascular diseases (dominant comparison)


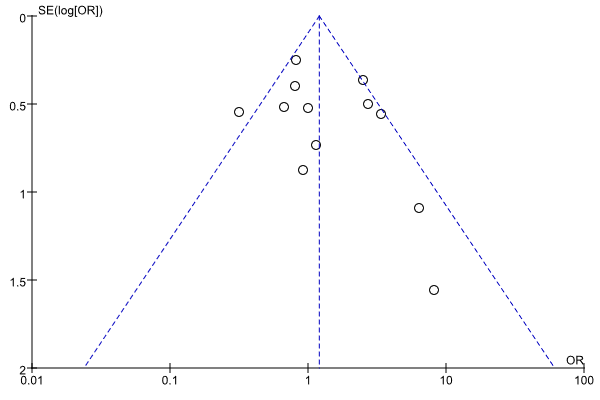


Funnel plot of rs1799983 polymorphism and hemorrhagic cerebral vascular diseases (recessive comparison)


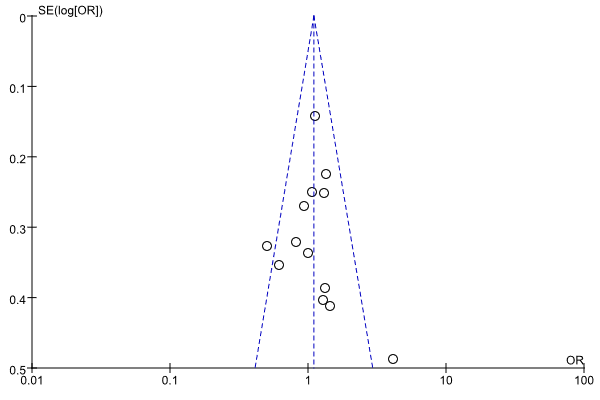


Funnel plot of rs1799983 polymorphism and hemorrhagic cerebral vascular diseases (over-dominant comparison)


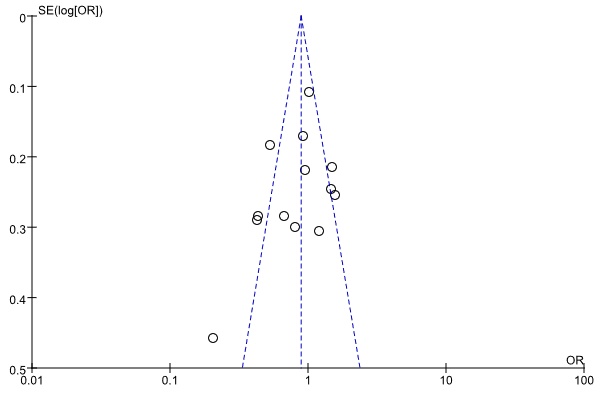


Funnel plot of rs1799983 polymorphism and hemorrhagic cerebral vascular diseases (allele comparison)


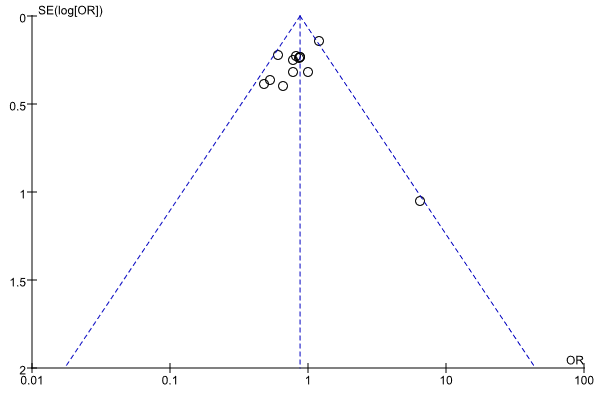
Funnel plot of rs2070744 polymorphism and hemorrhagic cerebral vascular diseases (dominant comparison)


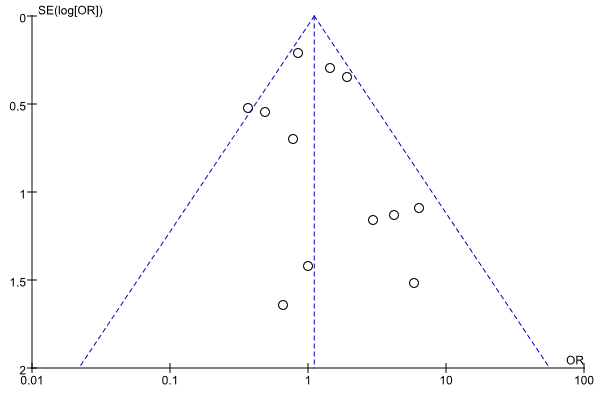


Funnel plot of rs2070744 polymorphism and hemorrhagic cerebral vascular diseases (recessive comparison)


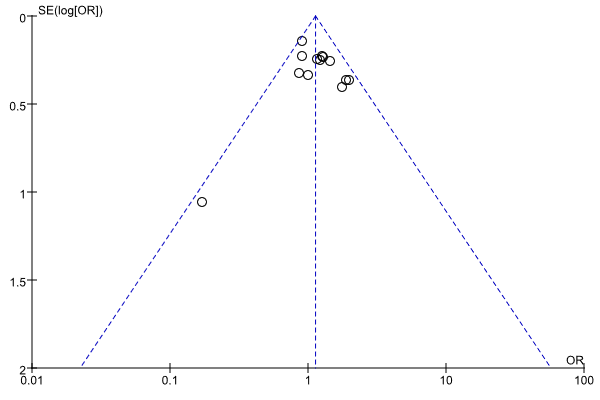


Funnel plot of rs2070744 polymorphism and hemorrhagic cerebral vascular diseases (over-dominant comparison)


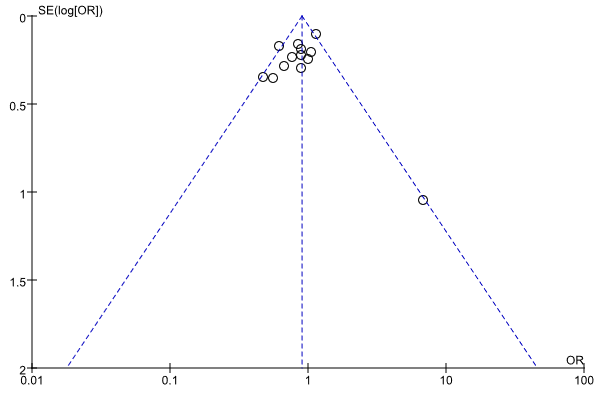


Funnel plot of rs2070744 polymorphism and hemorrhagic cerebral vascular diseases (allele comparison)


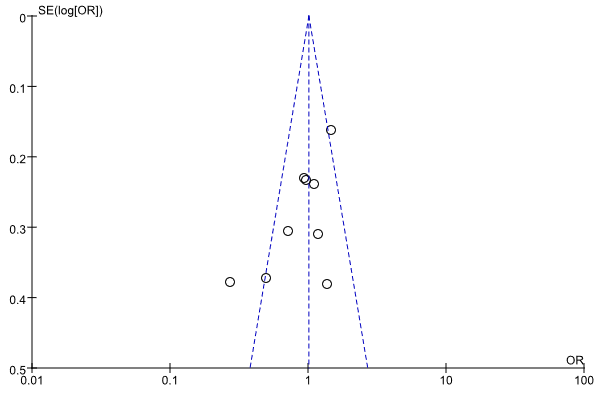
Funnel plot of rs869109213 polymorphism and hemorrhagic cerebral vascular diseases (dominant comparison)


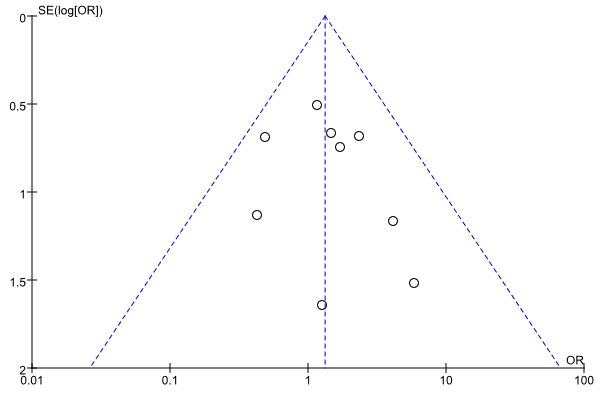


Funnel plot of rs869109213 polymorphism and hemorrhagic cerebral vascular diseases (recessive comparison)


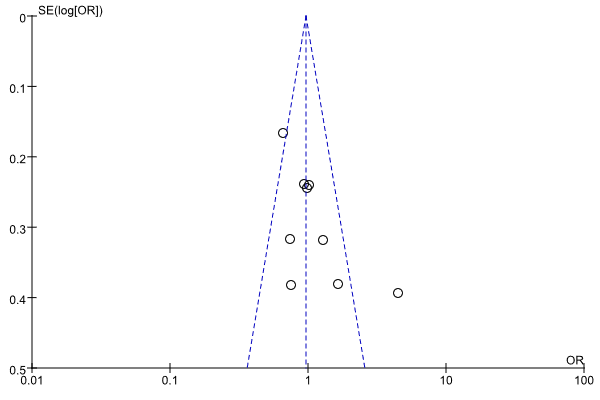


Funnel plot of rs869109213 polymorphism and hemorrhagic cerebral vascular diseases (over-dominant comparison)


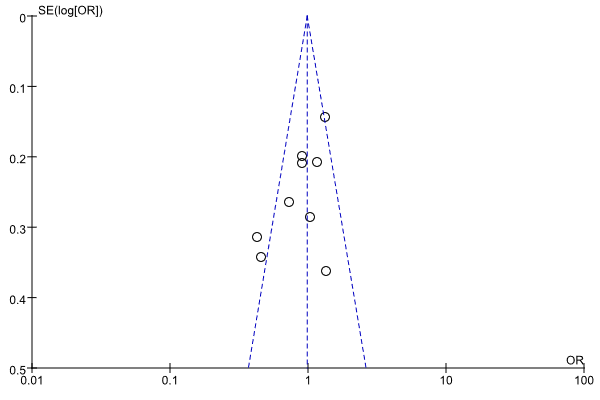


Funnel plot of rs869109213 polymorphism and hemorrhagic cerebral vascular diseases (allele comparison)
